# Point-of-care therapeutic drug monitoring of tumour necrosis factor-α inhibitors using a single step immunoassay†

**DOI:** 10.1039/d3sd00131h

**Published:** 2023-09-04

**Authors:** Eva A. van Aalen, Ivar R. de Vries, Eva T. L. Hanckmann, Jeannot R. F. Stevens, Thomas R. Romagnoli, Luc J. J. Derijks, Maarten A. C. Broeren, Maarten Merkx

**Affiliations:** a Laboratory of Chemical Biology, Department of Biomedical Engineering, Eindhoven University of Technology P.O. Box 513 5600 MB Eindhoven The Netherlands m.merkx@tue.nl +31 40 247 4728; b Institute for Complex Molecular Systems, Eindhoven University of Technology P.O. Box 513 5600 MB Eindhoven The Netherlands; c Department of Electrical Engineering, Eindhoven University of Technology P.O. Box 513 5600 MB Eindhoven The Netherlands; d Department of Clinical Pharmacy and Pharmacology, Máxima Medical Center P.O. Box 7777 5500 MB Veldhoven The Netherlands; e Department of Clinical Pharmacy and Toxicology, Maastricht University Medical Center P.O. Box 5800 6202 AZ Maastricht The Netherlands; f Laboratory of Clinical Chemistry and Haematology, Máxima Medical Center P.O. Box 7777 5500 MB Veldhoven The Netherlands

## Abstract

Therapeutic drug monitoring (TDM) of tumor necrosis factor-α (TNFα)-inhibitors adalimumab and infliximab is important to establish optimal drug dose and maximize treatment efficacy. Currently, TDM is primarily performed with ELISA techniques in clinical laboratories, resulting in a long sample-to-result workflow. Point-of-care (POC) detection of these therapeutic antibodies could significantly decrease turnaround times and allow for user-friendly home-testing. Here, we adapted the recently developed bioluminescent dRAPPID (dimeric Ratiometric Plug-and-Play Immunodiagnostics) sensor platform to allow POC TDM of infliximab and adalimumab. We applied the two best performing dRAPPID sensors, with limit-of-detections of 1 pM and 17 pM, to measure the infliximab and adalimumab levels in 49 and 40 patient serum samples, respectively. The analytical performance of dRAPPID was benchmarked with commercial ELISAs and yielded Pearson's correlation coefficients of 0.93 and 0.94 for infliximab and adalimumab, respectively. Furthermore, a dedicated bioluminescence reader was fabricated and used as a readout device for the TDM dRAPPID sensors. Subsequently, infliximab and adalimumab patient serum samples were measured with the TDM dRAPPID sensors and bioluminescence reader, yielding Pearson's correlation coefficients of 0.97 and 0.86 for infliximab and adalimumab, respectively, and small proportional differences with ELISA (slope was 0.97 ± 0.09 and 0.96 ± 0.20, respectively). The adalimumab and infliximab dRAPPID sensors, in combination with the dedicated bioluminescence reader, allow for ease-of-use TDM with a fast turnaround time and show potential for POC TDM outside of clinical laboratories.

## Abstract 2

Bioluminescent sensor proteins are reported for point-of-care drug monitoring of infliximab and adalimumab that rival the analytical performance of ELISA.

## Graphical abstract

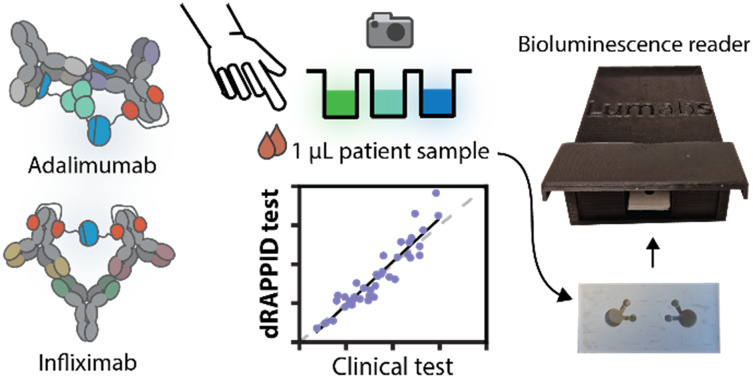

## Introduction

Infliximab and adalimumab are two anti-inflammatory drugs that target and neutralize the proinflammatory cytokine anti-tumour necrosis factor-α (TNFα). These therapeutic antibodies are widely administered for the treatment of diseases such as ulcerative colitis,^1^ Crohn's disease^2^ and rheumatoid arthritis.^3,4^ The precise dosing of infliximab and adalimumab is crucial to achieve the desired balance between drug efficacy and toxicity. However, the optimal dose of infliximab and adalimumab is highly patient-specific due to inter-patient variability in clearance rates, and can change over time if responsiveness to these drugs is reduced or lost as a result of anti-drug antibody (ADAb) formation.^5–11^ Hence, monitoring of adalimumab and infliximab levels can optimize treatment efficacy by determining the most appropriate individual dose.^12–16^

Currently, serum levels of infliximab and adalimumab are primarily determined with ELISA, the most widely used assay format in clinical laboratories for these analytes.^17^ Although this method is sensitive and allows for the detection of multiple samples in one ELISA run, it requires trained personnel, sophisticated incubation, washing and detection techniques, sample transport to the clinic and collection of multiple patient samples for an ELISA batch, engendering a long sample-to-result workflow. Point-of-care (POC) tests show great promise to decrease the turnaround time of current TDM and allow for more accessible and user-friendly TDM opportunities.^17^ Alternatives for ELISA have been developed, such as the lateral flow immunoassays (LFIAs) Quantum Blue® from Buhlmann and RIDA®QUICK by R-Biopharm.^18,19^ Although these tests are generally rapid, with turnaround times of 20 min, they still have some limitations such as the requirement of basic laboratory skills, separation of serum or plasma from blood, the need for relatively big sample volumes or quantitative differences with commercial ELISA kits, and are therefore less suitable for home-testing.^20–22^ In contrast, biosensors based on a bioluminescent readout are pertinent tools for simple homogeneous measurements in complex media,^23,24^ such as blood serum, and have demonstrated their potential for TDM POC applications.^25–29^ The recently developed dRAPPID (dimeric Ratiometric Plug-and-Play Immunodiagnostics) sensor format is based on analyte-induced split NanoLuc (NLuc) complementation.^30^ Binding of the protein of interest by analyte-specific antibodies, covalently coupled to the split NLuc domains by photo-cross-linkable protein G adapters, engenders the formation of a luminescent ternary complex.^31,32^ The mix-and-measure dRAPPID assay has a fast and simple sample-to-result workflow, requires low sample volumes (<10 μL) and, due to its stable ratiometric light output, allows for easy readout techniques and reliable analyte quantification.

**Fig. 1:**
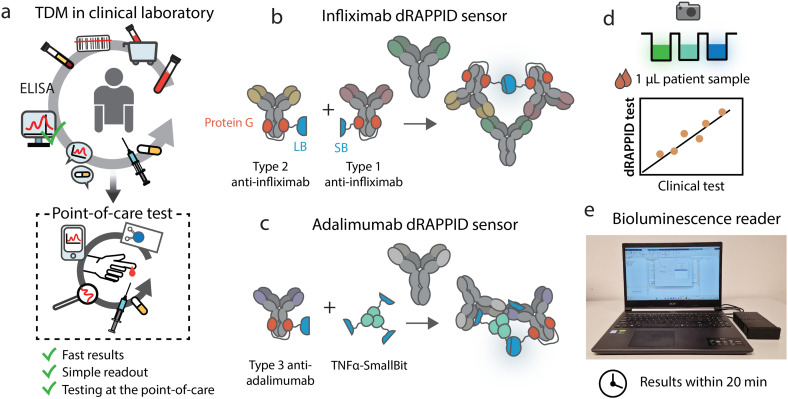
Development and implementation of the TDM dRAPPID sensors. (a) Translation of infliximab and adalimumab TDM from the clinical laboratory to a POC setting. Schematic overview of (b) the infliximab dRAPPID sensor and (c) the adalimumab dRAPPID sensor. Analyte binding results in the complementation of split NLuc and the emission of blue light. (d) The TDM dRAPPID sensors in (b) and (c) were applied to measure the infliximab and adalimumab concentrations in serum patient samples and results were compared with a clinical ELISA currently used in clinical laboratories. (e) The POC bioluminescence reader, dedicated to measure the blue-to-green ratios emitted by the infliximab and adalimumab dRAPPID biosensors.

Here, we apply the dRAPPID technology for the TDM of infliximab and adalimumab with the aim to translate TDM from the clinic to the POC (Fig. 1a). To this end, we screened and optimized different dRAPPID sensor combinations to obtain sensitive infliximab and adalimumab dRAPPID sensors, with >14-fold maximal increase in emission ratio (Fig. 1b and c). Importantly, we show that the two best performing dRAPPID sensors can be applied to measure therapeutic drug concentrations directly in 1 μL serum samples and display linear correlations with a clinical standard ELISA assay when tested in >40 patient samples (Fig. 1d). Finally, to increase the POC potential of the TDM dRAPPID platform, we developed an inexpensive, portable bioluminescence reader (Fig. 1e). With this reader, we quantified the concentration of adalimumab and infliximab in patient serum samples within 20 minutes, demonstrating the great potential of dRAPPID and the bioluminescence reader for TDM at the POC.

## Experimental

### Human subject statement

The protocol was cleared from approval by the local Medical Ethics Review Committee and the study was carried out in accordance with the Helsinki Declaration and Good Clinical Practice. All patient samples were collected as part of routine clinical care and anonymized before use in this study.

### Protein expression

Gx-d2-SB, Gx-d2-LB, TNFα–SB and the split calibrator luciferase (DNA and amino acid sequence in Fig. S1†) were expressed as described in ref. 28, 30 and 33.

### Photoconjugation

Anti-infliximab antibodies (HCA233 and HCA215) and anti-adalimumab (HCA2017), all from Bio-Rad, were preincubated for 45 minutes with Gx-d2-SB or Gx-d2-LB (1 μM antibody with 2 μM sensor) in PBS (pH 7.4). Subsequently, mixtures were placed under a UV lamp (Thorlabs M365LP1 with a Thorlabs LEDD1B T-Cube LED Driver) for 15 minutes. Photoconjugated sensors were not further purified and stored at 4 °C until use (Fig. S2†).

### dRAPPID assays in buffer

The SB-sensor (10 nM) was mixed with the LB-sensor (1 nM) and infliximab (from Máxima Medical Centre pharmacy in Veldhoven, the Netherlands) or adalimumab (cat# KBIY4001, Gentaur) in buffer (PBS (pH 7.4), 0.1% (w/v) BSA) and incubated for 45 minutes before the luminescent signal was recorded with a Tecan Spark 10 M plate reader or a digital camera (Sony DSC-Rx100 III). Measurements were performed in a volume of 20 μL in nontreated white Thermo Scientific 384-well plates (Cat. No. 262360) with 750-fold diluted NLuc substrate (Promega, N1110). For ratiometric measurements, the split calibrator luciferase, 10 pM for infliximab dRAPPID and 22 pM for adalimumab dRAPPID, was added and after 1 hour of incubation the blue-to-green ratios were calculated by dividing the light emission at 458 nm by the intensity at 518 nm. The LODs were calculated using eqn (1), in which SD is the standard error of the y-intercept, by linear regression of the response related to the analyte concentration for a limited range of concentrations.1
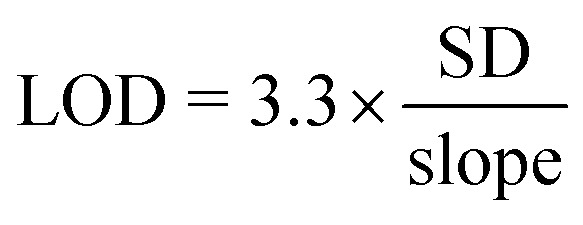


### dRAPPID with patient samples

Infliximab and adalimumab patients serum samples (1 μL) were diluted to a final 1000-fold or 500-fold dilution, respectively, in buffer (PBS (pH 7.4), 0.1% (w/v) BSA). dRAPPID sensors (1 nM LB, 10 nM SB and split calibrator) were added to the diluted patient samples. For calibration, infliximab or adalimumab standards (triplicates) were prepared and mixed with the same sensor batch used with the patient samples. Both patient samples and calibration curve were incubated for 1 hour at room temperature in 384-well plates. Subsequently, 750-fold diluted NLuc substrate was added and the luminescent signal was captured with a digital camera (integration time = 30 seconds, ISO value = 6400) in a light-tight Styrofoam box. Blue- and green light intensities of the samples were obtained (in predefined squares in the middle of each well) by analysing the pictures in ImageJ 1.51j8 software.^34^ The known log infliximab or adalimumab concentrations of the calibration curves were plotted against the obtained blue-to-green ratios. Next, a linear curve was fit through the linear part of the calibration curve data. This curve was subsequently used to translate the blue-to-green ratios of the patient samples to infliximab or adalimumab concentrations. ELISA measurements in the clinical laboratory were performed with an DS2® automated ELISA system (Dynex technologies) with commercially available ELISA kits for infliximab and adalimumab (Apdia, Turnhout, Belgium) according to the manufacturers protocol.

### Bioluminescence reader and disposable cartridges

The measurement circuit and design of the bioluminescence reader is illustrated in Fig. S3.† The three layers of the disposable cartridges were designed in AutoCAD software and cut using a laser cutter (VLS 3.50) from 1 mm thick transparent acrylate substrate (Fig. S4†). The three layers were subsequently assembled using double sided tape.

### dRAPPID with bioluminescence reader

10 nM of the LB sensor and 20 nM of the SB sensor were mixed with 90 pM or 180 pM split calibrator for infliximab or adalimumab, respectively, and 350-fold diluted NLuc substrate. These sensor mixtures were added to adalimumab or infliximab samples and incubated for 15 minutes for infliximab and 20 minutes for adalimumab in Eppendorf tubes. Subsequently, 50 μL of these mixtures were added to the disposable cartridges and blue-to-green ratios were measured with the bioluminescence reader. Infliximab and adalimumab patient serum samples (1 μL) were diluted 350-fold or 250-fold, respectively, and calibrations curves were used to obtain the concentrations of infliximab and adalimumab in the patient serum samples (Fig. S5†).

### Statistical analysis

Passing–Bablok regression analysis, performed in Analyse-it (Microsoft Excel), was used to compare the results of the dRAPPID (mean) with the ELISA. Bland–Altman analysis was performed in GraphPad Prism 7.00 and used to determine the agreement between the dRAPPID (mean values) and the apDia ELISA. Deming linear regression (GraphPad Prism 7.00) was applied to determine the relationship between the mean results with the bioluminescence reader dRAPPID and the ELISA.

## Results and discussion

### Development of sensors for infliximab and adalimumab

**Fig. 2:**
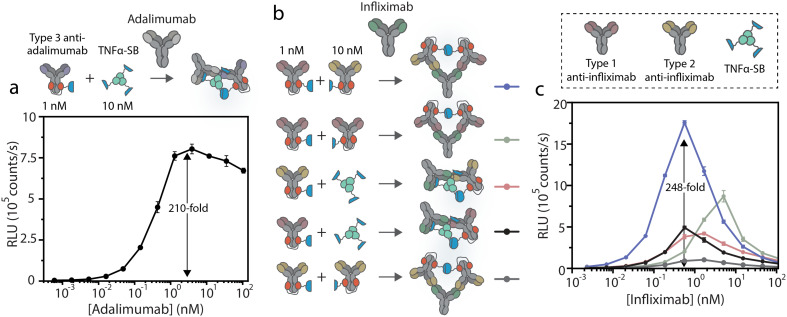
Performance of the adalimumab and infliximab dRAPPID assay. (a) Performance of the intensiometric adalimumab dRAPPID assay with 1 nM antibody-LB and 10 nM TNFα–SB and 45 minutes of incubation. (b) Schematic representation of the infliximab sensors that were screened for their performance. The assays were performed with 1 nM antibody-LB and 10 nM SB with 400-fold diluted NLuc substate, which was added after 45 minutes of incubation. (c) Intensiometric sensor output of the different infliximab assays. RLU, relative luminescence units. Values in (a) and (c) depict mean ± s.d. of technical replicates with independent preparations of target antibody.

We applied and adapted the recently established bioluminescent dRAPPID platform to realize a homogeneous sensor for infliximab and adalimumab TDM, with a simple read-out that allows applications in POC testing.^30^ The dRAPPID sensors consist of two analyte-specific binders connected to either a Large Bit (LB) or a Small Bit (SB, *K*_D_ = 2.5 μM) domain, the two fragments of split NLuc.^31^ In the presence of the furimazine substrate of NLuc and upon analyte-induced reconstitution of split NLuc, blue light is emitted. In the dRAPPID platform, the LB and SB fragments are connected *via* a semiflexible linker to the photo-cross-linkable dimeric protein G adapters (Gx-d2), giving rise to Gx-d2-SB and Gx-d2-LB sensor domains.^32^ To develop an adalimumab assay, we applied the previously reported combination of a type 3 anti-adalimumab antibody with a genetic fusion of TNFα to SB (TNFα–SB, Fig. 1c).^28^ The type 3 antibody in this assay binds the complex of TNFα(–SB) and adalimumab, reducing the amount inactive complexes arising from the binding of two SB or two LB-containing sensor components to the same target antibody. Accordingly, we conjugated the type 3 anti-adalimumab antibody to the Gx-d2-LB sensor domain and combined it with TNFα–SB (Fig. 1c and S2†). Next, we added 1 nM of the antibody-LB conjugate and 10 nM TNFα–SB to increasing concentrations of adalimumab, incubated this sensor-analyte mixture for 45 minutes, and subsequently added the substrate of NLuc to measure the resulting luminescent signal using a plate reader. This single-step immunoassay platform does not require washing steps, as the interaction of adalimumab with the sensor components (TNFα and the idiotypic antibody) facilitates the complementation of NLuc and the emission of blue light. Fig. 2a shows that this dRAPPID sensor yielded a 210-fold maximal increase in blue light emission at ∼3 nM adalimumab. The increase in blue light emission confirmed the binding of the sensor components to adalimumab and the ensuing formation of the luminescent ternary complex, yielding a limit-of-detection (LOD) of ∼2.6 pM (Fig. S6a†).

Unfortunately, no type 3 antibody was available for infliximab. Therefore, we developed and screened the performance of different types of infliximab dRAPPID assays to select a sensor that enabled sensitive infliximab detection and displayed a large change in blue light emission. To this end, we site-specifically photoconjugated the dRAPPID sensor domains (Gx-d2-SB or Gx-d2-LB) to the heavy chains of a type 1 anti-infliximab antibody, which recognizes free infliximab, and a type 2 anti-antibody, binding to both free and TNFα-bound infliximab (Fig. 1b and S2†). Besides the use of anti-idiotypic antibodies to capture infliximab, we also included TNFα–SB in the screening process.^28^ By combining the three analyte-binders we obtained and tested five different infliximab dRAPPID assay formats (Fig. 2b). Addition of increasing concentrations of infliximab to 1 nM of the LB-sensor domain and 10 nM of the SB-sensor protein displayed an infliximab-dependent increase in blue light emission for all sensor combinations (Fig. 2c). The best sensor combination, comprised of a type 1 anti-infliximab-LB and a type 2 anti-infliximab-SB, yielded a 248-fold maximal change in blue light emission at an infliximab concentration of ∼400 pM and displayed a LOD of ∼2.4 pM (Fig. S6b†). When we increased the infliximab concentration further, the blue luminescent signal decreased sharply due to the “hook” effect as a result of sensor components binding distinct infliximab proteins. The “hook” effect was less prominent for the adalimumab sensor compared to the infliximab sensors, which might be due to the presence of a relatively high concentration of the SB component in the former assay (30 nM, three TNFα proteins per SB protein).^28,35^

**Fig. 3:**
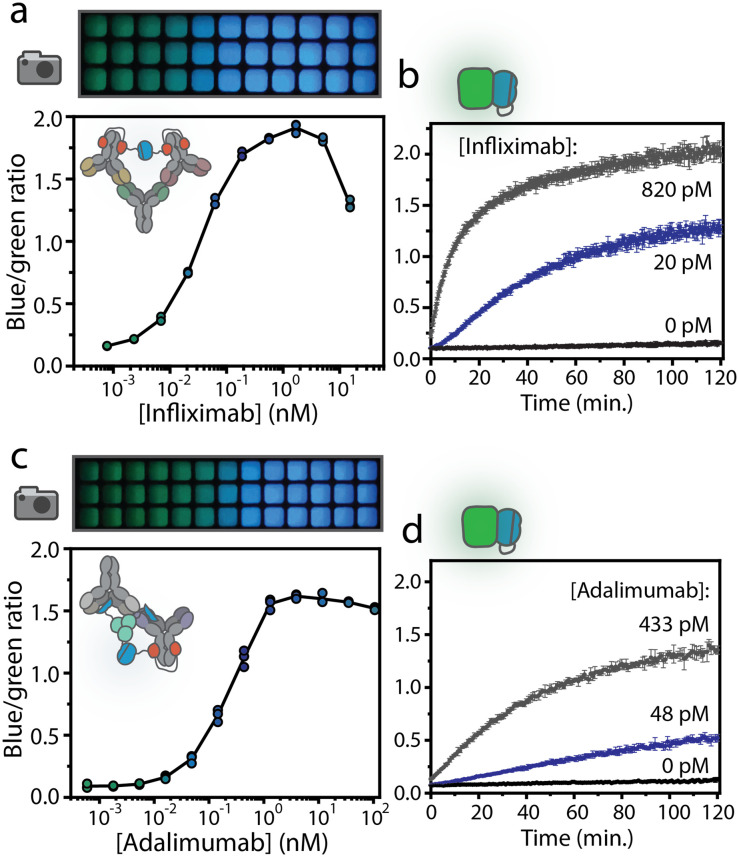
Ratiometric adalimumab and infliximab dRAPPID. (a) Ratiometric response of the best performing infliximab sensor, comprising of a type 1- and type 2 anti-infliximab antibody, with 10 pM of the split calibrator luciferase. The blue-to-green ratios in the graph were calculated by dividing the blue light intensity (458 nm) by the green light intensity (518 nm) measured with a plate reader. The picture above the graph was made with a digital camera. (b) Blue-to-green ratios over time at three different infliximab concentrations. Sensor components, infliximab and the substrate of NLuc were added at *t* = 0. (c) Ratiometric sensor output of the adalimumab dRAPPID. Analysis of the blue-to-green ratios was performed both with a plate reader (graph) and a digital camera (picture). (d) Ratiometric adalimumab-dependent response of the adalimumab dRAPPID over time after one-step incubation of all the dRAPPID components, with 22 pM of the split calibrator luciferase. Data points in (a) and (c) represent technical replicates, with *n* = 3 independent preparations of target antibody. Values in (b), and (d) depict mean ± s.d. of technical replicates.

For POC application, where non-experts handle measurements and environmental factors cannot be accurately controlled, a robust assay with a stable signal over time is required. Intensiometric dRAPPID assays, where blue light intensities correlate to analyte concentration, suffer from substrate depletion, resulting in changes in signal over time (Fig. S7†). Therefore, we next translated the best performing intensiometric infliximab and adalimumab dRAPPID sensors into ratiometric assays. We previously developed the green light-emitting calibrator luciferase, comprising of a genetic fusion of mNeonGreen en NLuc.^28,36,37^ By adding the calibrator luciferase to the sample and by dividing the blue light of the dRAPPID assay by the green light of the calibrator luciferase, stable ratiometric signals over time were achieved. However, the kinetics of substrate conversion by the calibrator luciferase and the reconstituted dRAPPID sensors are not identical, since this calibrator luciferase comprises of a full sized NLuc protein, whereas the dRAPPID platform is based on split NLuc complementation. This difference in enzyme kinetics can result in a decrease in ratiometric signal over time, especially for situations where high sensor concentrations are desired (Fig. S8a†). Accordingly, to reach stable ratiometric signals over time also in dRAPPID assays with high sensor concentrations, we adapted the previously developed calibrator luciferase by replacing the full size NLuc by the split version of NLuc (split calibrator luciferase). In this way, the kinetics of substrate conversion are the same for both the calibrator and the sensor, hence resulting in a more stable signal over time (Fig. S8b†). Fig. 3a shows the ratiometric response of the infliximab sensor with the new split calibrator luciferase. Increasing the concentration of infliximab resulted in an increase in blue-to-green ratio, observed both with plate reader analysis and in a photograph taken with a simple digital camera (Sony DSC-Rx100 III). The infliximab dRAPPID assay yielded a LOD of ∼1 pM and a 14.6-fold maximal change in emission ratio (Fig. S6c†). The ratiometric adalimumab sensor also exhibited a dose–response curve, with a maximal change in emission ratio (14.5-fold) at ∼3 nM and a LOD of ∼17 pM (Fig. 3c and S6d†). For both assays, the kinetics of complex formation of the dRAPPID with target antibody was slow at sub-nanomolar concentration of target antibody, reaching a stable signal after ∼100 minutes (Fig. 3b and d).

### Comparing the TDM dRAPPID with commercial ELISAs

**Fig. 4:**
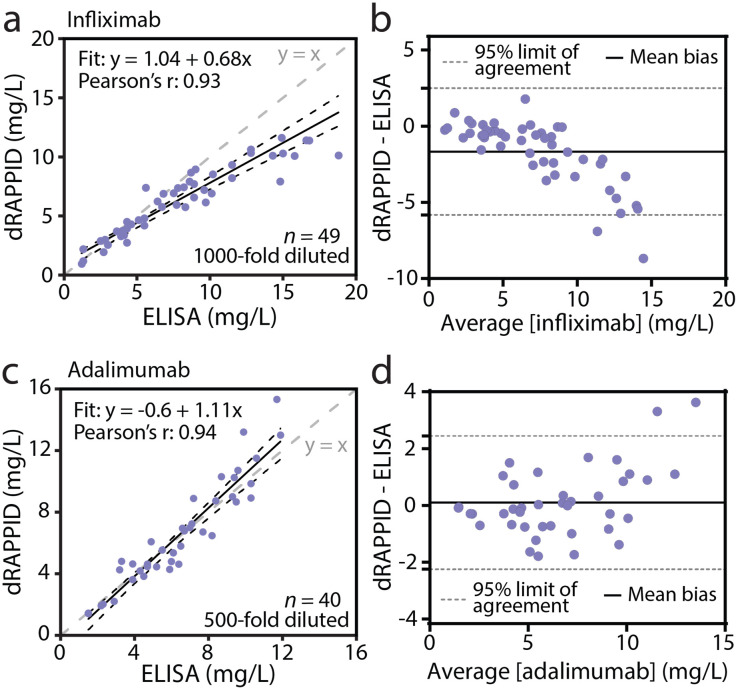
Comparison of dRAPPID with a clinical ELISA. (a) Passing–Bablok regression analysis of the infliximab dRAPPID and ELISA. (b) Bland–Altman analysis of the data described in panel a. (c) Passing–Bablok regression analysis of the adalimumab dRAPPID and ELISA. (d) Bland–Altman analysis of the data described in panel c. Values measured with the dRAPPID in (a) and (c) represent mean of technical replicates (*n* = 3).

To demonstrate the potential of the infliximab and adalimumab dRAPPID assays for TDM, we applied the two sensors to measure patient serum samples and compared their analytical performance with an automated ELISA assay used in the clinical laboratory. Due to the high sensitivity of the two dRAPPID sensors, the relevant therapeutic window of infliximab (20–134 nM) and adalimumab (33–81 nM) are both higher than the responsive range of the sensors (Fig. 3a and c).^38,39^ Therefore, dilution of the sample is necessary, allowing tunable measurements across the relevant antibody concentration range. Furthermore, dilution of samples with a complex matrix like whole blood or serum, reduces interference and hence increases sensitivity and specificity. We collected 1 μL infliximab patient serum samples (*n* = 49), diluted them 1000-fold in buffer (PBS (pH 7.4), 0.1% (w/v) BSA) and measured them using the ratiometric dRAPPID sensor. To capture the concentration-dependent blue-to-green ratios emitted by the TDM dRAPPID sensors, we used a simple measurement set-up of a digital camera in a Styrofoam box (Fig. S9†). Parallel to dRAPPID measurements, we quantified the same patient samples with the automated ELISA used in the clinic. Fig. 4a and S10a† show that the infliximab dRAPPID assay exhibited a linear correlation with the automated ELISA (Pearson's correlation coefficient = 0.93). However, for high infliximab concentrations, we consistently measure lower infliximab levels with our sensor, yielding a negative bias (−1.67 mg L^−1^ mean bias, Fig. 4b). This bias might be because the peak of the bell-shaped curve is reached at high infliximab concentrations, no longer allowing for reliable infliximab quantification above ∼10 mg L^−1^. To tune the detectable concentration range to higher infliximab concentrations, a larger dilution factor could be applied. Next, we compared the performance of the adalimumab dRAPPID assay with the automated ELISA. We diluted 1 μL adalimumab serum samples (*n* = 40) 500-fold with sensor components and buffer and captured the blue-to-green ratios with a digital camera. The adalimumab dRAPPID yielded a linear correlation (Pearson's correlation coefficient = 0.94) with the clinical ELISA (Fig. 4c and S10b†). Furthermore, Fig. 4d demonstrates a small proportional bias (mean bias: 0.102 mg L^−1^) between the two diagnostic methods, suggesting good agreement between adalimumab dRAPPID and the apDia ELISA.

### Development of a dedicated bioluminescence reader

**Fig. 5:**
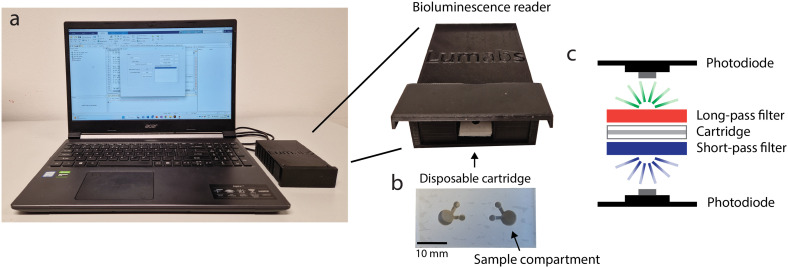
Overview of the bioluminescence reader setup. (a) The reader connected to a laptop showing the MATLAB graphical user interface. (b) The disposable cartridge which may be used for two independent measurements. (c) A schematic outline of the optical filtering and detection with photodiodes.

The use of a digital camera for measuring bioluminescence in 384-wellsplates provides a good alternative for more sophisticated plate reader-based measurements. However, for measurements at the POC, a cartridge with a stand-alone reader is more user-friendly and more robust than a digital camera. Therefore, we developed an easy-to-use, cheap and portable bioluminescence reader optimized for ratiometric detection of dRAPPID assays. This reader (Fig. 5a) was designed to capture bioluminescence efficiently with immediate display of the result, significantly improving the POC potential of the TDM dRAPPID sensors. The reader leverages a disposable cartridge (Fig. 5b) with a transparent top and bottom, which enables the emission of light on both sides of the cartridge, and two sample compartments to enable duplo measurements. This cartridge is placed between optical short- and long-pass filters, which both have a cut-off wavelength of 490 nm since the emissions of dRAPPID and the split calibrator peak at 460 nm (blue) and 517 nm (green) respectively. Hence, the blue and green light can be detected on separate sides of the cartridge. Subsequent efficient capturing of the green and blue light is done with two identical photodiodes that are placed very close to both sides of the transparent sample compartments of the cartridge (Fig. 5c and S3†). Next, the blue and green intensities are send to a personal computer *via* tailor-made hardware and the resulting blue-to-green ratio is subsequently calculated and displayed with a MATLAB Graphical User Interface.

### Point-of-care therapeutic drug monitoring with the bioluminescence reader

**Fig. 6:**
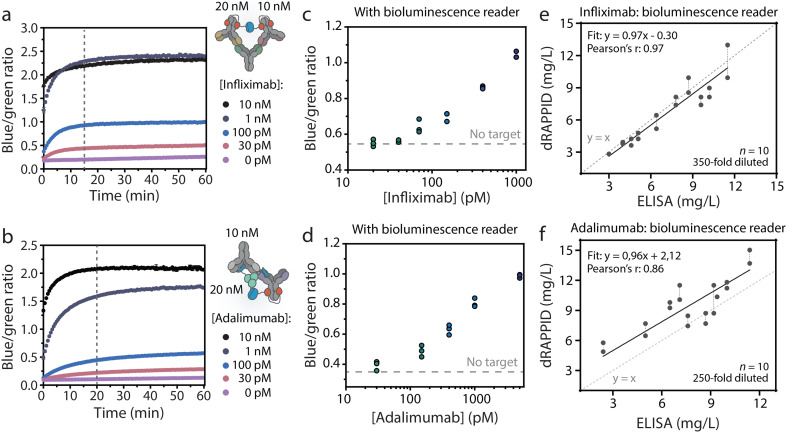
Detection of adalimumab and infliximab with the bioluminescence reader. Luminescence over time with (a) infliximab dRAPPID or (b) adalimumab dRAPPID sensor (10 nM LB with 20 nM SB). Blue-to-green ratios of (c) infliximab and (d) adalimumab captured with the reader (15 minutes of incubation) in buffer. Detection of (e) infliximab and (f) adalimumab in patient serum samples with dRAPPID in combination with the bioluminescence reader. The black lines in (e) and (f) represent Deming linear regression. Vertical dotted lines connects duplicate measurements of one patient sample. Values in (a) and (b) depict mean ± s.d. of technical replicates and data points in (c) and (d) represent technical replicates, with *n* = 3 independent preparations of target antibody.

The dRAPPID experiments reported so far contained 1 nM of the LB sensor component and 10 nM of the SB domain, which allowed sensitive quantification of the target antibody but required an incubation step of ∼1 hour to allow efficient complex formation between target and sensor. In POC detection, results should ideally be obtained within ∼20 minutes. Hence, we increased the concentration of the sensor components to 10 nM and 20 nM, for the LB component and SB component respectively, to speed up analyte binding (Fig. S11† for titrations). Furthermore, by increasing the sensor concentration we decreased the necessary dilution factor to reach the relevant concentration range. The plate reader analysis in Fig. 6a and b shows that increasing the infliximab and adalimumab sensor concentrations resulted in a stable signal after ∼15 minutes of incubation. With these increased sensor concentrations and the short incubation step, we measured different infliximab levels using the bioluminescence reader. To this end, we preincubated the sensor and infliximab for 15 minutes, injected 50 μL of this mixture into the disposable cartridge and measured the corresponding blue-to-green ratio with the bioluminescence reader. Fig. 6c shows that an infliximab-dependent increase in blue-to-green ratio was measured and that ∼70 pM infliximab could be discriminated from the background signal. Similarly, we applied the bioluminescence reader to measure different adalimumab concentrations in buffer and with only 20 minutes of incubation (Fig. 6d). As expected, an adalimumab-dependent increase in blue-to-green ratio was observed, illustrating the successful combination of dRAPPID and reader.

To demonstrate that the bioluminescence reader can be used for TDM in patient samples, we measured 10 infliximab- and 10 adalimumab patient samples with the dRAPPID sensors and measured the corresponding blue-to-green ratios with the reader (Fig. S5†). Fig. 6e shows that measurements with the infliximab dRAPPID and the reader displayed an excellent correlation (Pearson's correlation coefficient = 0.97) and a small proportional difference (slope = 0.97 ± 0.09 and *y*-intercept = −0.30 ± 0.68) with the clinical ELISA. The adalimumab quantification with dRAPPID and the bioluminescence reader also yielded a linear correlation (Pearson's correlation coefficient = 0.86) with the ELISA and showed a slightly larger proportional difference (slope = 0.96 ± 0.20 and *y*-intercept = 2.12 ± 1.62) with ELISA than the infliximab sensor (Fig. 6f).

## Conclusions

The optimal dosage of infliximab and adalimumab is highly patient-specific and several studies have demonstrated that higher trough levels (*i.e.* above a certain acutoff value) are often associated with higher response and remission rates.^40–43^ Hence, therapeutic drug monitoring can contribute to precision dosing and improve efficacy of the treatment. Currently, serum concentrations of infliximab and adalimumab are often measured in the clinical laboratory using ELISA techniques. However, these clinical immunoassays entail a long sample-to-result workflow, expert operators and sophisticated detection techniques, and are thus not suited for drug monitoring in home settings. To this end, we have adapted the dRAPPID platform to obtain two robust sensors for the homogenous quantification of the therapeutic antibodies infliximab and adalimumab, showing great potential for POC detection. These dRAPPID sensors, in combination with a simple digital camera, were subsequently applied to measure drug concentrations in >40 serum patients samples and results were benchmarked with the apDia ELISA. Both TDM dRAPPID assays showed a linear correlation with the automated ELISA, with the adalimumab dRAPPID yielding a small proportional bias and the infliximab dRAPPID assay displaying a negative bias at higher infliximab concentrations. To decrease this bias at infliximab levels exceeding ∼10 mg L^−1^ and obtain results within the measuring range of the assay, samples could be further diluted with buffer. Altogether, these first results of the TDM dRAPPID platform with patient serum samples are promising and suggest a good agreement with the ELISA. However, to assess the full potential of the sensors for TDM, more patient samples should be measured, and the repeatability and intermediate precision of the sensors should be determined.

The TDM dRAPPID requires a very small sample volume of only 1 μL. Thus, a simple and non-invasive finger prick can be performed to obtain the required sample volume, significantly increasing the POC potential of the sensors. However, in this study we only tested the dRAPPID sensors on pre-treated (serum) patient samples. Although we found that haemolyzed samples did not impede measurements, in the future the performance of the sensors should be tested in whole blood samples to remove any sample-pre-treatment steps. Similar to most lateral flow immunoassays, the dRAPPID sensors require one short incubation step of ∼20 minutes and no washing steps. This allows for a much shorter sample-to-result workflow than the clinically used ELISA and immediate adjustment of drug dosage. Hence, the dRAPPID platform also shows potential for TDM in the clinical laboratory setting, significantly decreasing the turnaround times of the current TDM methods.

To bring the TDM dRAPPID platform closer to the POC, we designed and developed a dedicated bioluminescence reader with compatible disposable cartridges for duplo measurements. This simple, fast and portable device displays the results within 3 minutes and improved the potential of the dRAPPIDs for POC TDM. The current disposable cartridge still requires premixing of the sensors, NLuc substate and sample. In the future, we envision the development of a microfluidic chip with integrated sensor components and NLuc substrate. With this, the only required actions taken by the user would be to add the sample to the chip and insert it into the bioluminescence reader. A possible challenge of integrating the dRAPPID platform in POC microfluidic chips is the limited long-term stability of the sensor components, particularly the NLuc substrate. However, this issue could be resolved by adopting a recently reported protocol for creating a stable lyophilized all-in-one reagent.^44^ The herein developed POC TDM dRAPPIDs and the bioluminescence reader brings us one step closer to monitoring drug trough levels at home, facilitating the implementation of patient-specific precision dosing.

## Author contributions

E. V. A. designed the study, performed experiments, analysed the data and wrote the manuscript. I. D. V. designed and developed the bioluminescence reader and wrote the manuscript. E. H. contributed important initial work and established a proof of concept. J. S. and T. R. contributed to the design and development of the bioluminescence reader. L. D. and M. B. conceived and supervised the study with the patient serum samples and M. M. conceived, designed and supervised the study. All authors discussed the results and commented on the manuscript.

## Conflicts of interest

Maarten Merkx filed a patent application on 22 June 2020 on RAPPID and the ratiometric detection of luciferase assays using a calibrator luciferase (The Netherlands patent application PCT/NL2020/050406; patent applicant: Eindhoven University of Technology).

## Supplementary Material

SD-002-D3SD00131H-s001
