# Supplementary material for: Point-of-care therapeutic drug monitoring of tumour necrosis factor-α inhibitors using a single step immunoassay
Source: Sens Diagn. 2023 Sep 4;2(6):1492–500. doi: 10.1039/d3sd00131h (PMC10633107; doi:10.1039/d3sd00131h)
Supplement: SD-002-D3SD00131H-s001 [file SD-002-D3SD00131H-s001.pdf]

Electronic Supplementary Material (ESI) for Sensors & Diagnostics.
This journal is © The Royal Society of Chemistry 2023

Point-of-care therapeutic drug monitoring of tumour necrosis factor-α inhibitors using a single step immunoassay

Eva A. van Aalen[ab], Ivar R. de Vries[c], Eva T. L. Hanckmann[ab], Jeannot R. F. Stevens[ab], Thomas R. Romagnoli[ab], Luc J. J. Derijks[de], Maarten A. C. Broeren[f] and Maarten Merkx*[ab]

[a] Laboratory of Chemical Biology, Department of Biomedical Engineering, Eindhoven University of Technology, P.O Box 513, 5600 MB Eindhoven, The Netherlands.
[b] Institute for Complex Molecular Systems, Eindhoven University of Technology, P.O Box 513, 5600 MB Eindhoven, The Netherlands.
[c] Department of Electrical Engineering, Eindhoven University of Technology, P.O Box 513, 5600 MB Eindhoven, The Netherlands.
[d] Department of Clinical Pharmacy and Pharmacology, Máxima Medical Center, PO Box 7777, 5500 MB Veldhoven, the Netherlands.
[e] Department of Clinical Pharmacy and Toxicology, Maastricht University Medical Center, PO Box 5800, 6202 AZ Maastricht, The Netherlands.
[f] Laboratory of Clinical Chemistry and Haematology, Máxima Medical Center, PO Box 7777, 5500 MB Veldhoven, the Netherlands.

Content

		Page
Figure S1	DNA and amino acid sequence of the split calibrator luciferase	S2
Figure S2	Photoconjugation of anti-infliximab and anti-adalimumab	S3
Figure S3	Measurement circuit and design of the bioluminescent reader	S3
Figure S4	Fabrication of the disposable cartridges	S4
Figure S5	Calibration curves for measurements with the bioluminescent reader	S4
Figure S6	Limit-of-detection (LOD) of the TDM dRAPPID sensors	S5
Figure S7	Intensiometric infliximab and adalimumab over time	S6
Figure S8	Comparing the split calibrator luciferase with the full calibrator	S7
Figure S9	Measurement set-up with the digital camera	S7
Figure S10	Comparison of dRAPPID with the ELISA	S8
Figure S11	Dose-response curves with higher sensor concentration	S8

```
 M  G  S  S  H  H  H  H  H  H  S  S  G  L  V  P  R  G  S  H
ATGGGCAGCAGCCATCATCATCATCATCACAGCAGCGGCCTGGTGCCGCGCGGCAGCCAT
 M  V  S  K  G  E  E  D  N  M  A  S  L  P  A  T  H  E  L  H
ATGGTAAGTAAAGGTGAAGAAGACAATATGGCTTCTCTGCCTGCCACACATGAGCTTCAT
 I  F  G  S  I  N  G  V  D  F  D  M  V  G  Q  G  T  G  N  P
ATTTTTGGGAGCATAAACGGAGTGGATTTCGACATGGTAGGTCAGGGTACGGGGAACCCT
 N  D  G  Y  E  E  L  N  L  K  S  T  K  G  D  L  Q  F  S  P
AACGATGGATATGAGGAGTTGAATCTTAAAAGCACAAAGGGTGATCTGCAGTTCTCGCCC
 W  I  L  V  P  H  I  G  Y  G  F  H  Q  Y  L  P  Y  P  D  G
TGGATCCTGGTGCCGCATATAGGTTATGGTTTCCATCAGTATCTTCCATACCCGGATGGC
 M  S  P  F  Q  A  A  M  V  D  G  S  G  Y  Q  V  H  R  T  M
ATGAGCCCTTTTCAGGCCGCAATGGTAGATGGCTCAGGATATCAAGTGCATCGGACCATG
 Q  F  E  D  G  A  S  L  T  V  N  Y  R  Y  T  Y  E  G  S  H
CAGTTTGAAGATGGGGCGTCTTTGACGGTAAATTACAGGTACACCTATGAGGGTAGCCAT
 I  K  G  E  A  Q  V  K  G  T  G  F  P  A  D  G  P  V  M  T
ATAAAGGGAGAAGCGCAGGTGAAGGGAACTGGATTCCCAGCGGATGGCCCAGTCATGACA
 N  S  L  T  A  A  D  W  C  R  S  K  K  T  Y  P  N  D  K  T
AACAGCCTCACCGCTGCTGATTGGTGCCGATCCAAGAAAACGTATCCAAACGATAAAACT
 I  I  S  T  F  K  W  S  Y  T  T  G  N  G  K  R  Y  R  S  T
ATCATTTCTACTTTTAAGTGGTCCTATACAACAGGAAACGGGAAACGCTATCGTTCAACG
 A  R  T  T  Y  T  F  A  K  P  M  A  A  N  Y  L  K  N  Q  P
GCCCGCACGACCTACACGTTTGCAAAGCCAATGGCTGCGAATTATCTGAAAAACCAGCCG
 M  Y  V  F  R  K  T  E  L  K  H  S  K  T  E  L  N  F  K  E
ATGTATGTGTTCCGTAAAACCGAACTGAAACATTCTAAAACGGAGCTCAATTTCAAGGAA
 W  Q  K  A  F  T  G  F  E  D  F  V  G  D  W  E  Q  T  A  A
TGGCAGAAGGCATTTACCGGTTTTGAAGATTTCGTTGGGGACTGGGAACAGACAGCCGCC
 Y  N  L  D  Q  V  L  E  Q  G  G  V  S  S  L  L  Q  N  L  A
TACAACCTGGACCAAGTCCTTGAACAGGGAGGTGTGTCCAGTTTGCTGCAGAATCTCGCC
 V  S  V  T  P  I  Q  R  I  V  R  S  G  E  N  A  L  K  I  D
GTGTCCGTAACTCCGATCCAAAGGATTGTCCGGAGCGGTGAAAATGCCCTGAAGATCGAC
 I  H  V  I  I  P  Y  E  G  L  S  A  D  Q  M  A  Q  I  E  E
ATCCATGTCATCATCCCGTATGAAGGTCTGAGCGCCGACCAAATGGCCCAGATCGAAGAG
 V  F  K  V  V  Y  P  V  D  D  H  H  F  K  V  I  L  P  Y  G
GTGTTTAAGGTGGTGTACCCTGTGGATGATCATCACTTTAAGGTGATCCTGCCCTATGGC
 T  L  V  I  D  G  V  T  P  N  M  L  N  Y  F  G  R  P  Y  E
ACACTGGTAATCGACGGGGTTACGCCGAACATGCTGAACTATTTCGGACGGCCGTATGAA
 G  I  A  V  F  D  G  K  K  I  T  V  T  G  T  L  W  N  G  N
GGCATCGCCGTGTTCGACGGCAAAAAGATCACTGTAACAGGGACCCTGTGGAACGGCAAC
 K  I  I  D  E  R  L  I  T  P  D  G  S  M  L  F  R  V  T  I
AAAATTATCGACGAGCGCCTGATCACCCCCGACGGCTCCATGCTGTTCCGAGTAACCATC
 N  G  S  G  G  S  G  G  S  G  G  S  G  G  S  G  G  S  G  T
AACGGATCTGGTGGCTCAGGTGGTAGTGGAGGATCAGGAGGTAGTGGCGGATCAGGTACC
 V  T  G  Y  R  L  F  E  K  E  S  G  G  S  H  H  H  H  H  H
GTTACCGGCTATCGTCTGTTTGAAAAAGAGAGCGGTGGTTCACATCATCACCATCACCAC
```

Figure S1. DNA and amino acid sequence of the split calibrator luciferase. His-tag (red), mNeonGreen (green), Large BiT (dark blue) and SmallBiT (light blue).

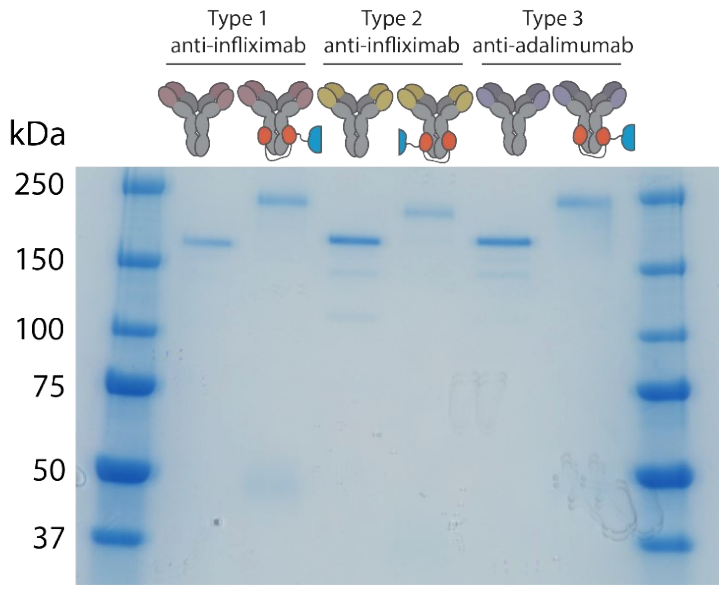

Figure S2. Non-reducing SDS-PAGE analysis of the photo-conjugations of the TDM dRAPPID sensors. 1 μM antibody was mixed with 2 μM sensor protein (Gx-d2-SB or Gx-d2-LB) in PBS (pH 7.4) and incubated for 45 minutes. Subsequently, the mixture was irradiated with UV light for 15 minutes.

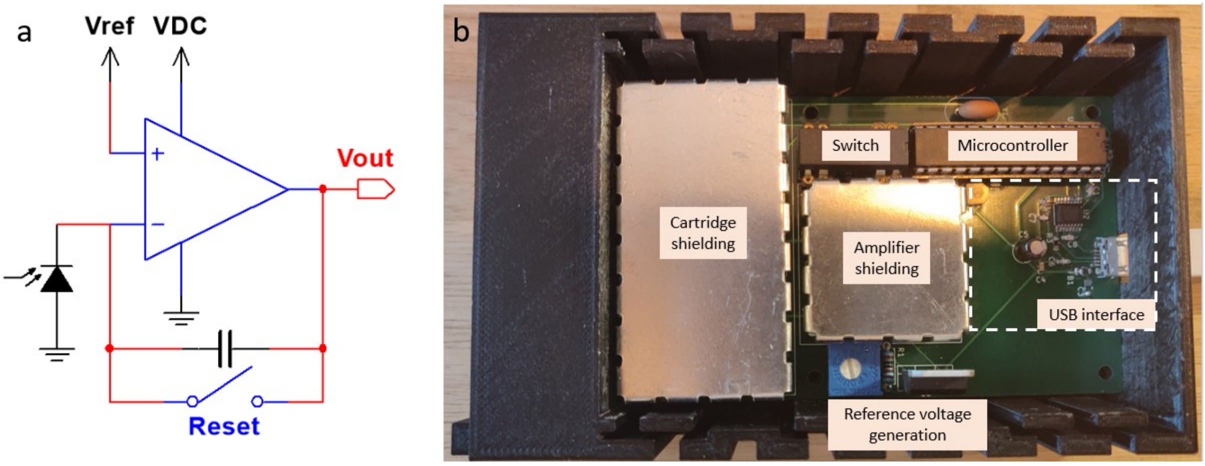

Figure S3. Measurement circuit and design of the bioluminescent reader. (a) the integrator circuit where an operational amplifier in combination with a capacitor is used to integrate the current generated by the photodiode in response to light. A microcontroller (ATMega328p) is used to operate the reset switch and read the output voltage. The reference voltage Vref is manually set with an LM317 voltage regulator. (b) Top view of the interior of the bioluminescent reader, where a cartridge is inserted on the left. Steel shielding is used to limit the influence of light and other electromagnetic interference to the photodiodes and amplifier circuits. The housing was designed to limit self-heating of the device while minimizing light interference and was 3D printed with acrylonitrile butadiene styrene.

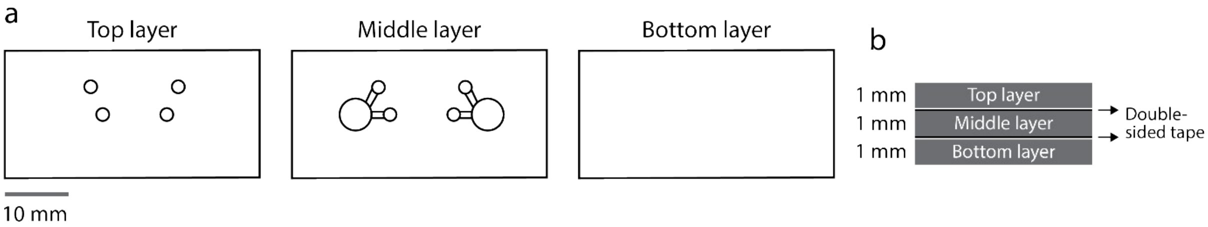

Figure S4. Fabrication of the disposable cartridges. (a) The three different layers of the chip. The layers were designed using AutoCAD software and a laser cutter was used to cut the desired features into 1 mm thick acrylate substrate. Two wells were added to one chip, allowing duplo measurements of the same sample. (b) Assembly of the disposable chip. Prior to laser cutting, double-sided tape (3M) was added to both sides of the middle layer to allow easy assembly of the chip by simply removing the protective layer of the double-sided tape and applying pressure on the three layers.

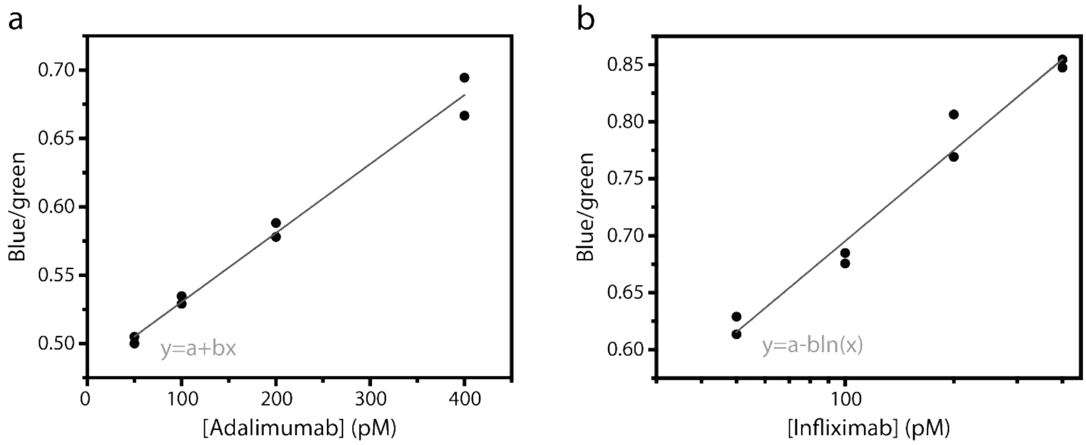

Figure S5. Calibration curves for measurements with the bioluminescent reader. (a) dRAPPID adalimumab calibration curve, with 10 nM LB and 20 nM SB and different concentrations of adalimumab. (b) dRAPPID infliximab calibration curve, with 10 nM LB-component and 20 nM SB-component with 4 different infliximab concentrations. Infliximab or adalimumab concentrations in patient samples were obtained by comparing blue-to-green ratios from the samples with the fit (black lines) in the graphs and extracting corresponding target concentrations.

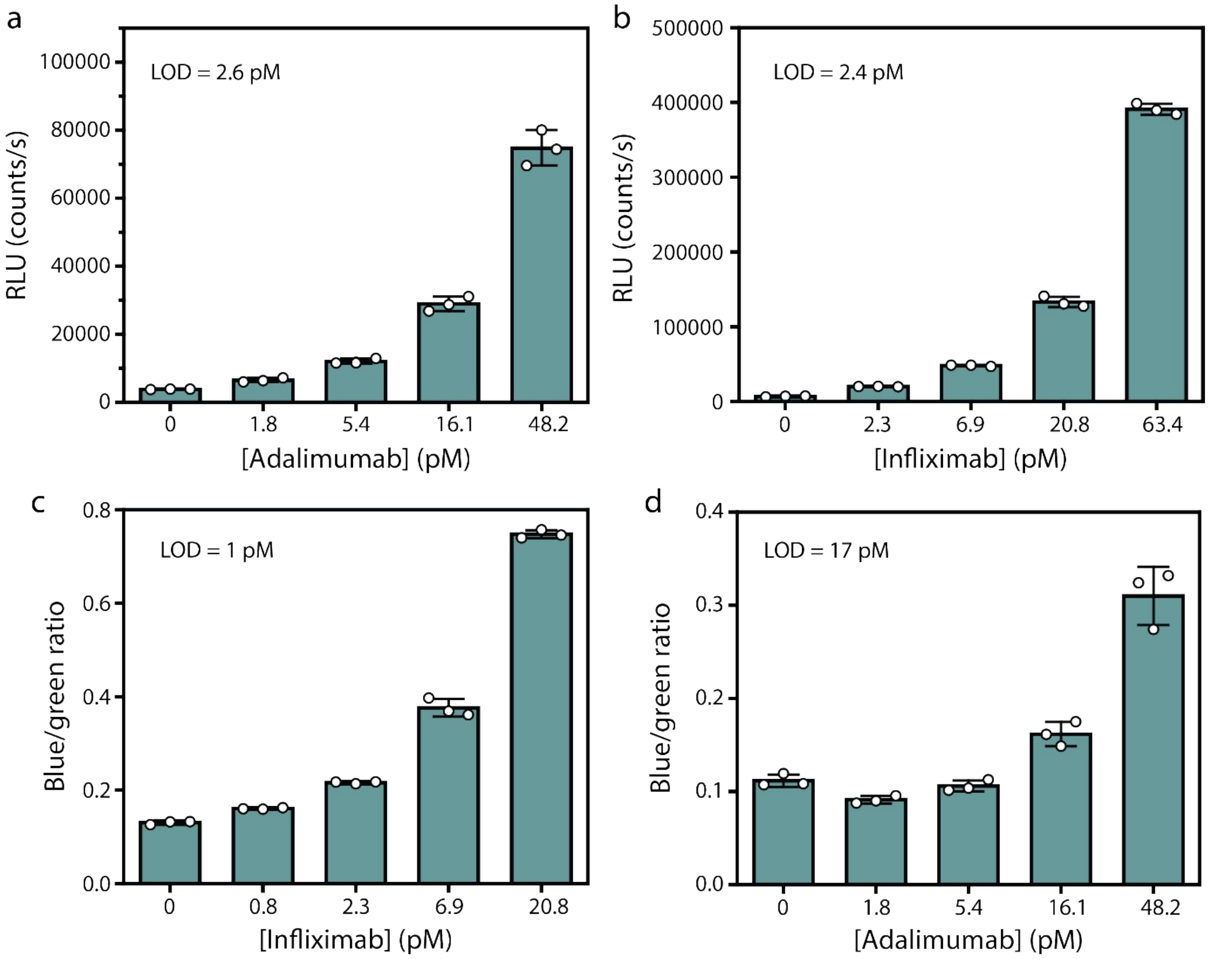

Figure S6. Limit-of-detection (LOD) measurement of the TDM dRAPPID sensors in Figure 2 and 3. LOD determination of the intensiometric (a) adalimumab dRAPPID (type 3 antibody with TNFα-SB) and (b) infliximab dRAPPID (type 1 and type 2 antibody). LOD determination of the ratiometric (c) infliximab dRAPPID with 10 pM split calibrator and (d) adalimumab dRAPPID with 22 pM split calibrator. For all sensors 1 nM of the LB component and 10 nM of the SB component was used. Measurements were done in buffer (PBS (pH 7.4), 0.1% (w/v) BSA). Data points represent technical replicates, with $n = 3$ independent preparations of target analyte.

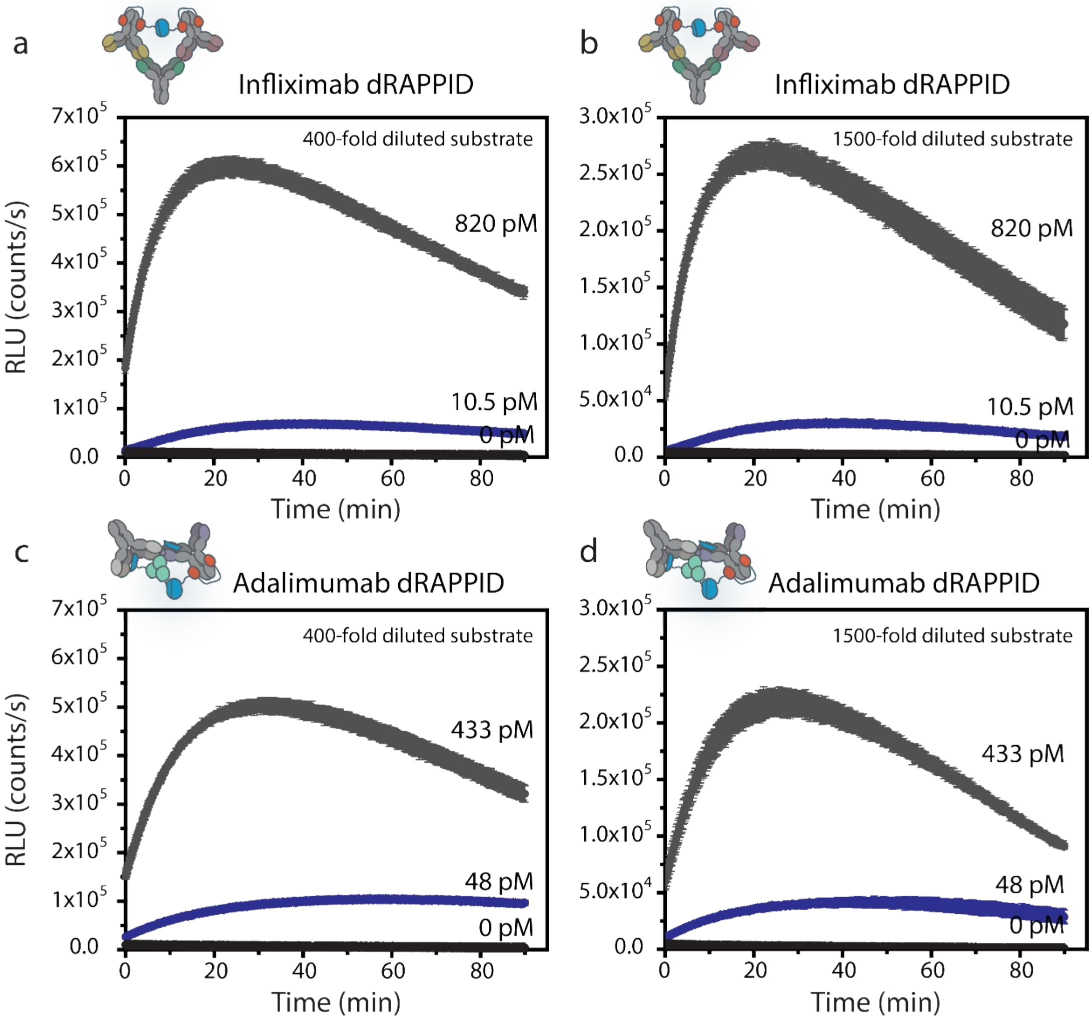

Figure S7. Intensiometric infliximab and adalimumab dRAPPID assays over time. Intensiometric sensor output of the infliximab dRAPPID with (a) 400-fold and (b) 1500-fold diluted NLuc substrate. Intensiometric blue light signal of the Adalimumab dRAPPID sensor with (c) 400-fold and (d) 1500-fold diluted NLuc substrate. All components (1 nM LB-sensor, 10 nM SB-sensor, substrate of NLuc and target antibody) were added at t=0.

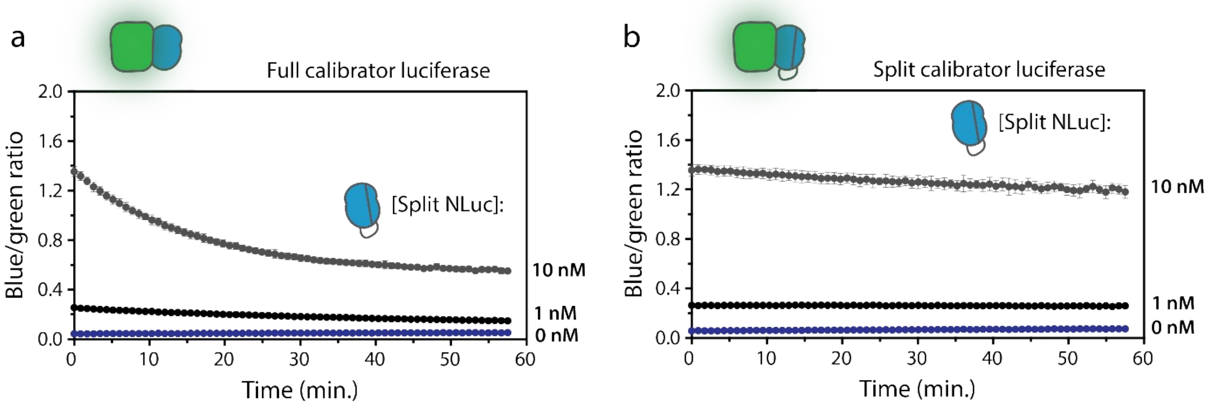

Figure S8. Comparing the split calibrator luciferase with the full calibrator luciferase. (a) Blue/green ratio of the full calibrator over time. Different concentrations of a split NLuc fusion protein (LB-linker-SB) were incubated with 185 pM of the full size calibrator luciferase and 400-fold diluted substrate of NLuc. (b) Ratiometric output of 500 pM of the split calibrator with different concentrations of split NLuc (10 nM, 1 nM and 0 nM) and 400-fold diluted NLuc substrate.

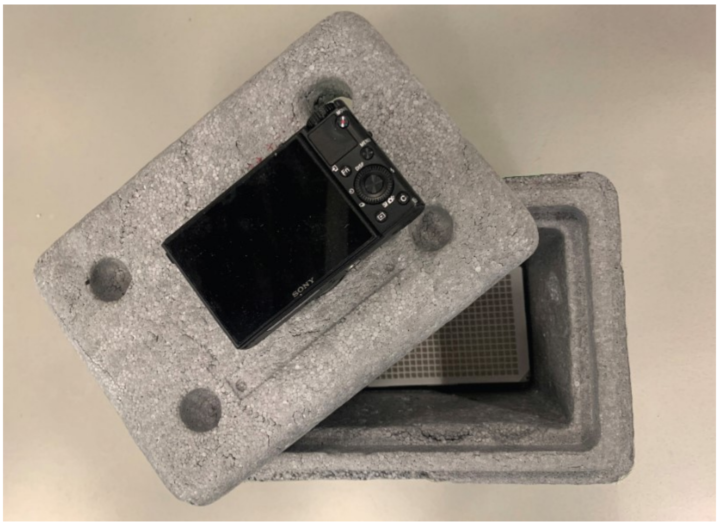

Figure S9. Measurement set-up with the digital camera (Sony DSC-Rx100 III) and Styrofoam box. A 384-wells plate was placed inside the light-tight box and pictures of the bioluminescence were taken with an integration time of 30 seconds and an ISO value of 6400.

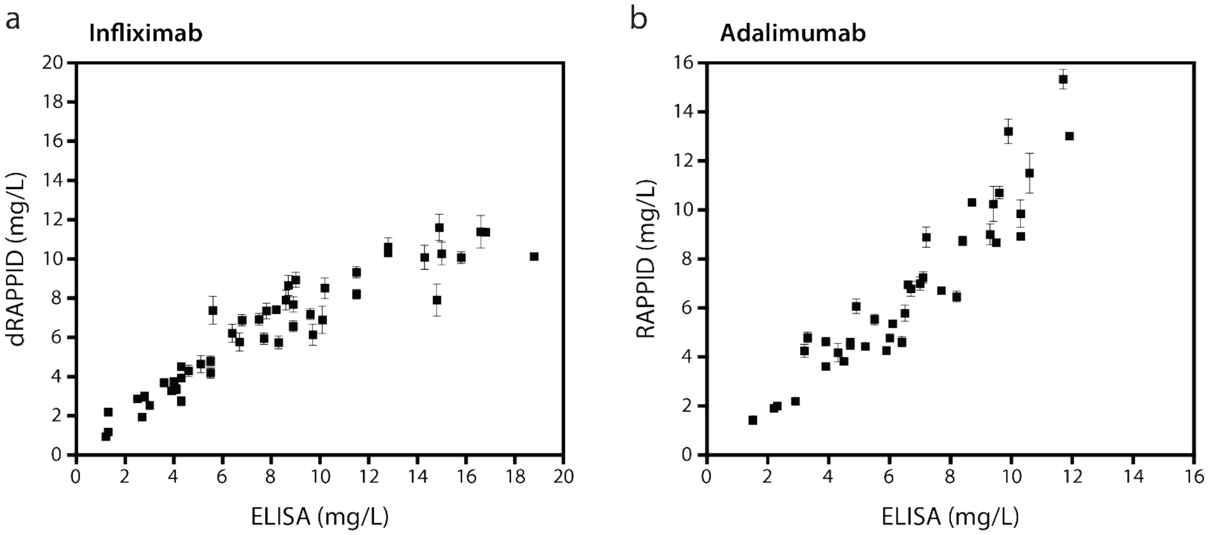

Figure S10. Comparison of dRAPPID with ELISA. (a) Infliximab dRAPPID assay plotted against ELISA. (b) Adalimumab dRAPPID results plotted against the ELISA results. Values depict mean ± s.d. of technical replicates.

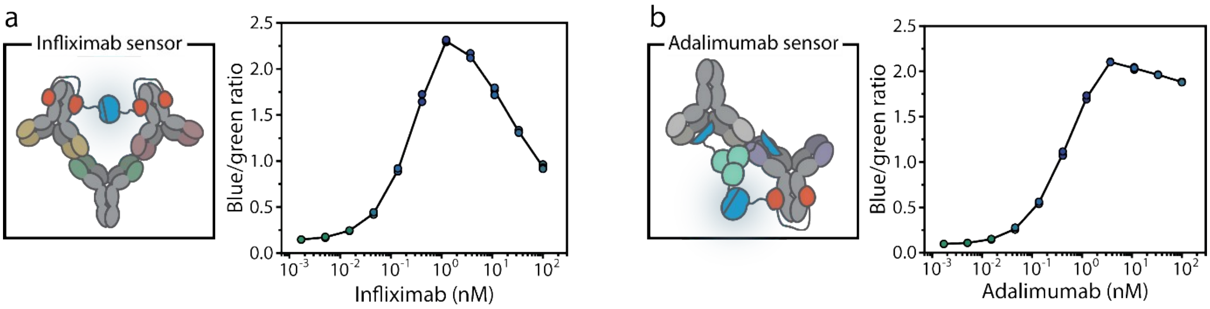

Figure S11. Dose-response curves of the (a) infliximab and (b) adalimumab dRAPPID with higher sensor concentrations and 96 pM or 210 pM split calibrator luciferase, respectively. 10 nM LB-sensor and 20 nM SB-sensor were mixed with target antibody and incubated for 1 hour at room temperature. Subsequently, 500-fold diluted NLuc substrate was added and bioluminescence was measured with a plate reader.
